# Citrus Pulp as an Alternative Energy Source for High-Yielding Dairy Cows in Tropical Systems: Effects on Intake, Digestibility, Nitrogen Balance, and Dairy Performance

**DOI:** 10.3390/ani16050806

**Published:** 2026-03-05

**Authors:** Elmer Edgardo Corea Guillen, Gabriela Alejandra Flores Leiva, Manuel Vicente Mendoza, Aurora Hilda Ramirez-Perez, Augusto Cesar Lizarazo, Nelson Alirio Cruz, Joaquín Miguel Castro-Montoya, Ever del Jesus Flores Santiago, Juan Carlos Ángeles-Hernandez

**Affiliations:** 1 Programa de Maestría y Doctorado en Ciencias de la Salud y Producción Animal, Universidad Nacional Autónoma de México, Mexico City 04510, Mexico; 2 Departamento de Zootecnia, Facultad de Ciencias Agronómicas, Universidad de El Salvador, San Salvador 1101, El Salvador; gabriela.leiva@ues.edu.sv (G.A.F.L.); manuel.mendoza@ues.edu.sv (M.V.M.); 3 Departamento de Nutrición Animal y Bioquímica, Facultad de Medicina Veterinaria y Zootecnia, Universidad Nacional Autónoma de México, Mexico City 04510, Mexico; 4 Centro de Enseñanza Práctica e Investigación en Producción y Salud Animal (CEPIPSA) Facultad de Medicina Veterinaria y Zootecnia, Universidad Nacional Autónoma de México, Mexico City 04510, Mexico; 5 Departament de Ciències Matemàtiques i Informàtica, Universitat de les Illes Balears, 07122 Palma, Spain; neacruzgu@unal.edu.co; 6 Instituto de Ciencias Agroalimentarias y Ambientales, Facultad de Ciencias Agronómicas, Universidad de El Salvador, San Salvador 1101, El Salvador; 7 Unidad Regional Universitaria Sursureste—Universidad Autónoma Chapingo, Texcoco 56230, Mexico; 8 Departamento de Medicina y Zootecnia en Rumiantes, Facultad de Medicina Veterinaria y Zootecnia, Universidad Nacional Autónoma de México, Mexico City 04510, Mexico

**Keywords:** cattle, dairy, tropical climate, byproduct

## Abstract

This study evaluated the impact of incorporating citrus pulp (CiP) into the rations of high-producing dairy cows under tropical conditions. Eighteen lactating dairy cows were assigned to two dietary treatments: corn meal (CM) or CiP as the main energy source. Dairy cows were allocated to a crossover design comprising two 21-day periods. The rations were formulated to be isocaloric and isonitrogenous. Replacing CM with CiP reduced the intakes of dry matter (19.9 vs. 19.5 kg/d), organic matter (17.9 vs. 17.4 kg/d), digestible organic matter (12.3 vs. 11.7 kg/d), and crude protein (3.43 vs. 3.35 kg/d), while increasing neutral detergent fibre intake (7.39 vs. 7.63 kg/d). Apparent total tract digestibility decreased for all nutrients, including DM, OM, CP, NDF, and ADF, when CiP replaced CM. Milk production was lower in cows fed CiP than in those fed CM (23.7 vs. 22.7 kg/d), although milk feed efficiency (milk/DMI) was not different. An economic analysis showed that cows fed CM had higher milk gross income and income over feed cost. These results suggest that the partial replacement (60%) of CM with CiP may negatively affect feed intake, nutrient digestibility, milk production, and profitability in dairy cows in tropical regions.

## Simple Summary

The aim of this study was to evaluate the effects of replacing dietary corn meal with pelleted citrus pulp on nutrient use, microbial protein synthesis, and performance in dairy cows in tropical conditions. The inclusion of citrus pulp reduced the intake and digestibility of nutrients, the capture of nitrogen by microbes, milk yield and income over feed cost, but not the efficiency of feed-to-milk conversion. Nevertheless, due to its availability, price and inedibility for humans, citrus pulp can be a suitable option as a source of nutrients in dairy cow production systems in tropical regions

## 1. Introduction

The increase in the global population over the last century has intensified the demand for high-quality food derived from healthy and sustainable animal production systems [1]. Among farm livestock, dairy cattle are the most efficient animals in converting feed protein and energy into food [2]. However, when fed human-edible feeds such as grains and pulses, dairy cows become inefficient in transforming these resources into animal products; furthermore, ration costs become higher with their use [3].

The efficient use and expansion of a wide variety of feed resources, as well as the identification of new feeds that do not compete with human food, are essential to meet the demand for animal feed to promote sustainable livestock production [4]. Agro-industrial byproducts are an important source of alternative feeds, as materials often considered waste can be used as cost-effective energy sources in ruminant rations [5]; including them in livestock feed offers advantages: a) it reduces the animals’ dependence on human edible grains, and b) it reduces the costs of their disposal and mitigates their environmental impact [6]. In this context, citrus pulp (CiP) has been identified as a highly suitable byproduct due to its high availability, and its nutritional composition indicates that it could help meet the energy needs for maintenance, growth, reproduction, and production in ruminants [6,7].

The term “citrus pulp” encompasses the waste generated during the processing of citrus fruits such as oranges, lemons, limes, grapefruits, mandarins, and kinnows. Around 30% of global citrus production, and up to 40% of orange production, is used for juice production [8]. This process generates large amounts of waste; CiP, composed of peel, pulp, and seeds, represents almost 50% of the processed fruit mass [9]. CiP is used as an important ingredient in ruminant rations in many parts of the world [7].

Citrus fruit pulp contains significant amounts of pectin, a structural carbohydrate in the plant cell wall [10]. It is widely used as a cattle feed ingredient; however, results in dairy cow rations have been inconsistent [11,12]. The chemical composition and physical characteristics of CiP can vary considerably depending on the fruit species and the processing methods used [7]. Dehydrated CiP is commonly used as a grain replacer in diets for lactating dairy cows due to its high organic matter digestibility (850–900 g/kg DM), crude protein content (6.16–6.70 g/100 g DM), metabolizable (ME) and net (NE) energy availability (11.6–12.1 MJ/kg DM and 6.94–7.36 MJ/kg DM); the ME content of CiP is comparable to that of barley, representing approximately 85–90% of the ME value of maize [6,7]. Thus, the use of citrus pulp provides an alternative to the use of cereals (rich in starch) in the rations of dairy cows fed a high proportion of concentrate [12], especially in tropical regions where CiP is readily available at a lower cost than imported maize, the main energy source.

Several studies have evaluated the inclusion of CiP in the diets of dairy cows, generally supporting its value as a partial replacement for cereal grains and as a viable energy source in concentrate mixtures [6,10]. Reported benefits include improved diet digestibility and feed efficiency, as well as greater cost-effectiveness compared with conventional grain-based diets [13,14]. However, most of the existing research has been conducted in temperate production systems, while there is limited evidence of the use of Cip in tropical dairy systems. Therefore, this study aimed to evaluate the effects of including dried citrus pulp in the rations of dairy cows under tropical conditions on intake, nutrient utilisation, nitrogen balance, microbial protein synthesis, and milk and nutrient yields.

## 2. Materials and Methods

### 2.1. Location

This experiment was conducted on a commercial dairy farm in Caluco, western El Salvador, at 13°43′ North Latitude and 89°40′ West Longitude, at 357 m above sea level. According to Holdridge’s [15] classification, the climate is classified as a dry tropical forest. The average annual rainfall is 1861 mm, with an average annual temperature of 25 °C. It is characterised by two well-defined seasons, a dry season from November to April and a rainy season from May to October [16].

### 2.2. Animals, Experimental Design and Feeding

This experiment lasted 42 days, divided into two periods: 15 days for adaptation and 6 days for sampling and data recording. Eighteen lactating Holstein cows with an average body weight (BW) of 536 ± 48.4 kg, 99 ± 46 days in milk (DIM), and 23.9 ± 3.5 kg of milk yield (MY) were used in the study under a cross-over design with those effects homogeneously distributed between treatments; at the end of the first experimental period, the cows were switched between treatments. The cows were housed in two adjacent pens with zinc-sheet roofs and concrete floors; each pen had individual sand-lined resting places. The available feeder per animal was 1 m long. Treatment for heat stress was applied to cows by means of water sprinkles and fans of 70 cm diameter from 8:00 am to 5:00 pm. Milking was carried out twice, in the morning (3:00 am) and in the afternoon (3:00 pm) in a mechanised milking parlour.

Cows were randomly placed in one of the two experimental groups (9 cows per group) based on the dietary energy source—corn meal (CM) or citrus pulp (CiP) + CM—homogeneously distributed by BW, MY, and DIM. The CiP was purchased dehydrated from a local supplier. The two diets were isoenergetic and isonitrogenous formulated according to NRC [17] recommendations for dairy cows with 550 kg BW and 25 kg MY with a forage-to-concentrate ratio of 50:50 on a DM basis as follows: corn meal ration (CM; 200 g CM/kg DM) and citrus pulp ration (CiP; 120 g CiP + 80 g CM/kg DM). Both diets contained the same proportion of corn silage, napier grass (Pennisetum purpureum), Vigna hay (Vigna sinensis) and Swazi grass hay (Digitaria swazilandensis) and were offered as total mixed rations (TMR). Table 1 presents the chemical composition of the forages and the main energy sources (CM and CiP), while Table 2 shows the ingredients and composition of the complete diets. Feed intake was adjusted to an expected refusal of 5% of wet feed, and fresh drinking water was provided ad libitum.

### 2.3. Sampling and Data Collection

At the beginning and end of each experimental period, cows were weighed (BH60 cattle scale, Sipel S.R.L., Rosario, Argentina) on two consecutive days. On the six sampling days, the total amounts of feed offered and refused, as well as individual milk production, were recorded. In addition, 400 g/d of ingredients, forage, TMR, and orts were collected in plastic bags and stored at −20 °C for laboratory analysis.

Urine and faeces were collected daily from each cow at different times (d1, 07:00; d2, 09:00; d3, 11:00; d4, 13:00; d5, 15:00; d6, 17:00 h). Urination was stimulated using perineal massage to obtain 1000 mL of urine; 100 mL of urine was taken and acidified (sulfuric acid, 20% *v*/*v*) to lower the pH below 3 and filtered through Whatman No. 42 filter paper (Thomas Scientific, Swedesboro, NJ, USA). Subsequently, 10 mL of acidified and filtered urine was diluted with 50 mL of distilled water. From this dilution, three 10 mL plastic containers with screw caps were filled. Ten mL of undiluted acidified urine was collected for nitrogen (N) analysis. Faecal samples (200 g) were obtained directly from the rectum of each cow and collected in a plastic bag. Both urine and faeces were frozen (−20 °C) until laboratory analysis.

During the sampling periods, 50 mL of milk was taken directly from the milking machine from each cow twice a day every third day. These primary samples were placed in sterile plastic bags and refrigerated (4 °C). Once the six samples were collected, they were pooled and homogenised for shipment to the La Salud Cooperative’s milk laboratory for physicochemical analysis.

### 2.4. Laboratory Analysis and Calculations

In the laboratory, TMR, ingredients, and orts (200 g) were thawed; faecal samples were also pooled per cow per period. To determine DM, all materials (TMR, ingredients, ords, and faeces) were dried in a programmable forced-air oven (model OF-22P, JEIO TECH™, Daejeon, Republic of Korea) at 60 °C for 48 h. Faeces were dried in a fan oven (model 100–800, Memmert GmbH and Co. KG™, Schwabach, Germany) for 72 h. The dried materials were ground (Wiley™ mill, Arthur H. Thomas Company, Philadelphia, PA, USA) to a 1 mm particle size.

Nitrogen (N) content was obtained by the Kjeldahl method (Velp DK digestion and UDK distillation; VELP™ Scientifica, Usmate, Italy) and crude protein (CP) was estimated using a factor of 6.25 (AOAC method 990.13) [18]. It should be noted that the N content of faeces was determined in thawed wet samples. Ash and organic matter (OM) contents were obtained by incineration at 600 °C for 2 h, (L24/12/P320 muffle furnace, Nabertherm™, Bremen, Germany). Neutral detergent fibre (NDF) using thermostable α-amylase and acid detergent fibre (ADF), both with residual ash included, were determined using a fibre analyzer (Ankom™ 200 Fiber Analyzer, ANKOM Technology, Macedon, NY, USA) following the methods described by Van Soest et al. [19]. Using the DM, OM, CP, NDF, and ADF concentrations of feed and orts, the daily amounts of feed and nutrient intake were estimated as described by Corea et al. [20]: feed or nutrient offered minus feed or nutrient refused. Acid-insoluble ashes (AIA) were quantified in feeds and faeces according to Van Keulen and Young [21] to be used as an internal marker to estimate faecal output (kg DM/d), following Patel et al. [22]: AIA intake (g)/AIA faeces concentration (kg AIA/kg faeces DM).

With the data on nutrient intake (DM, OM, NDF, ADF) and faecal excretion of the same, the apparent total tract digestibility (ATTD) was calculated using the following equation:

ATTD (g/100 g ingested) = [(Nutrient intake (g) − Faecal nutrient excretion (g))/Nutrient intake (g)] × 100(1)

Urine samples were thawed, pooled by cow for each period, and homogenised for creatinine and uric acid quantification by colorimetry with kinetic assays (CREJ2 and UA2 kits, respectively; Roche Diagnostics, Mannheim, Germany) in a photometer (COBAS C 501 module, Roche Diagnostics, Mannheim, Germany). Urinary excretion (L/animal/d) was estimated from urinary creatinine, assuming a constant excretion of 29 mg/kg BW/d, following the procedure described by Valadares et al. [23].

Urine volume (L/d) = (BW (kg) × 29 mg)/Creatinine concentration (mg/L)(2)

Allantoin was quantified spectrophotometrically (Jenway 6305, Cole-Palmer, Stone Staffs, Loughborough, UK), following the procedures described by Chen and Gomez [24]. Urinary excretion of purine derivatives (PD, mmol/animal/d) was calculated by multiplying allantoin and uric acid concentrations (mmol/L) by urine volume (L/d). Ruminal microbial protein synthesis (MPS, g/d) was estimated from absorbed microbial purines [25], calculated as the sum of daily uric acid and allantoin excretions, corrected for the endogenous PD fractions in urine (0.385 mmol/ kg0.75 BW) and the absorbed microbial purine bases recovered in urine (0.85) [25]. Urinary nitrogen was also quantified to estimate daily N excretion.

Milk samples were analysed using a FOSS MilkoScan™ FT1 (Foss Electric. Hillerød, Denmark) for total solids, protein, fat, lactose, and milk urea nitrogen (MUN) concentrations. The cow’s milk nutrient yields (protein, fat, lactose, and total solids, kg/d) were obtained by multiplying the nutrient concentration (g/kg) by individual milk yield. Nitrogen excretion (urine, faeces, and milk, g/d) was estimated from the amount excreted in each case, multiplied by its respective N concentration.

### 2.5. Economic Comparison of Diets

The costs per kg of DM and daily ration (USD) were determined based on the inclusion and price of each ingredient in the rations. Income over feed cost (IOFC) was calculated by subtracting the cost of the ration consumed from the gross income from milk sales (0.72 USD/kg of milk).

### 2.6. Statistical Analysis

R-Studio (version 4.0.2; R Core Team, Vienna, Austria) was used to analyse the data with the CrossCarry package [26]. The effect of energy source (CM and CiP) on the response variables was analysed according to a crossover design. With n = 9, the statistical power of this design is guaranteed at least 90% for treatment differences greater than 0.25 units in the response variable [26]. The model for variables measured once per cow in each period (digestibility, milk nutrients, nitrogen balance, microbial protein synthesis, and economic comparison) as follows:

Y_ij_ = µ + P_i_ + Td_ij_ + Rd_j−1, j_ + βNP_j_ + e_ij_

where Y_ij_ corresponds to the observation of the cow i in the period j; µ, the overall mean of the response variable throughout the experiment; P_i_, the effect of the period (i = 1, 2); Td_ij_, the effect of the treatment applied in the period i to the cow j, (j = 1, …, n); Rd_j−1, j_, the carryover effect of the treatment applied in the previous period over the period i in the cow j; β, the slope of the parity effect NP_j_ of the cow j; e_ij_, the error that is independent among cows with constant variances and mean 0.

The model used for variables measured more than once (nutrient intake) in the period was as follows:

Y_ijk_ = µ + P_i_ + Td_ij_ + Rd_j−1, j_ + βNP_j_ + B_k_ + e_ijk_

where Y_ijk_ is the observation of the dependent variable in the cow i in the period j; µ, the overall mean of the response variable throughout the experiment; P_i_, the effect of the period (i = 1, 2); Td_ij_, the effect of the treatment applied in the period i to the cow j (j = 1, …, n); Rd_j−1, j_, the carryover effect of the treatment applied in the previous period over the period i in the cow j; β, the slope of the parity effect NP_j_ of the cow j; B_k_, the effect of measurement time k over the response; e_ijk_, the error that is independent among cows, with constant variances and mean 0. Statistical significance was set at *p* < 0.05 and trends at *p* < 0.10.

## 3. Results

The nutritional composition of forages and the main energy sources used in the rations are shown in Table 1, where differences between the intervention factors are evident, mainly associated with the higher NDF (278 vs. 106 g/kg) and ADF (201 vs. 25.3 g/kg) contents of CiP compared with CM. The overall diet composition is presented in Table 2. In the experimental diets, CiP replaced 60% of CM (from 200 to 80 g/kg DM) while maintaining the same forage composition. This substitution resulted in diets with similar CP and ME but with an increase of approximately 27 g/kg DM in NDF in the CiP diet, which could influence fibre intake and digestibility. The main differences between CM and CiP diets were likely related to the higher structural carbohydrate content of the CiP diet.

The results of nutrient intake and digestibility in the rations evaluated in the present study show that replacing CM with CiP resulted in reductions (*p* < 0.01) in nutrient intake (Table 3). DMI decreased from 19.9 to 19.5 kg/d, OM intake from 17.9 to 17.4 kg/d, DOM from 12.3 to 11.7 kg/d, and CP from 3.43 to 3.35 kg/d. In addition, NDF intake was higher in CiP diets but ADF intake remained similar between treatments (*p* = 0.14), suggesting that replacing CM with CiP primarily affected the soluble and slowly fermentable fibre fractions. The ATTD of all nutrients decreased (*p* < 0.05) in the cows with the substitution of CM with Cip.

The results of milk production, productive efficiency (kg milk/kg feed), chemical composition and daily production of nutrients in milk are presented in Table 4. CiP used as the primary energy source decreased milk production from 23.7 to 22.7 kg/d (*p* < 0.01) compared with CM. This indicates that, although energy source substitution decreased milk production, production efficiency per kg of feed ingested remained similar between cows fed the CiP and CM rations.

Milk composition showed reductions in protein (31.1 vs. 30.1 g/kg) and total solids (124 vs. 123 g/kg) in response to CiP inclusion, whereas fat and lactose remained unchanged. Consequently, nutrient yields (protein, fat, lactose, and total solids) were higher in the CM treatment (*p* < 0.05), reflecting the combined effect of higher intakes and digestibility. In addition, MUN was lower in CM cows (14.0 vs. 14.6 mg/dL; *p* < 0.05), indicating differences in N utilisation and protein metabolism between treatments (Table 4).

Nitrogen metabolism was influenced by diet type. N intake was higher in CM cows (550 vs. 538 g/d), as well as urinary N (262 vs. 240 g/d) and milk N (118 vs. 109 g/d; *p* < 0.01). Relative to N intake, CM cows excreted a higher proportion of N in urine and milk, while faecal N was lower (*p* < 0.05; Table 5). Excretion of PD and MPS (1842 vs. 1561 g/d) was higher (*p* < 0.01) in CM-fed cows, resulting in greater microbial N efficiency (microbial N/N intake).

Economic outcomes were slightly affected by diet composition. Daily feed cost was lower for CiP (5.28 vs. 5.51 US$/d); however, indicators of economic efficiency showed that the higher productivity of CM rations resulted in better overall returns. CM cows presented higher milk income and IOFC (17.07 vs. 16.30 and 11.57 vs. 11.03 US$/d; *p* < 0.05), as well as a lower cost-to-benefit ratio (0.50 vs. 0.56 feed cost/milk income; Table 6).

## 4. Discussion

### 4.1. Nutrient Intake

The replacement of 60% of corn meal with citrus pulp reduced DM, OM, DOM and CP but not NDF and ADF intakes. Although some studies have reported no effect of feeding CiP on nutrient intake in dairy cows [27,28], most have shown a decrease [12]. It has been suggested that higher contents of NDF and ADF in CiP play a role in reducing digestibility and passage rate as a cause of lower intake [29]. Although in the current study, the diets were balanced for energy, there were differences in the contributions of NDF and ADF between the CM and CiP rations, with higher structural carbohydrates in the Cip diet (NDF, 27; ADF, 19 g/kg DM; Table 2), which depends in part on the NDF (278 vs. 106 g/kg) and ADF (201 vs. 25.3 g/kg) concentrations of CiP and CM, respectively (Table 1).

Pectin is an abundant heteropolysaccharide found in CiP (22–42 g/100 g) [11] that has been negatively associated with DMI in post-rumen infusion studies in dairy cows [30] and goats [31]. These studies showed that increases in abomasal pectin infusion resulted in linear decreases in DMI, which could be partially attributed to increased reticulorumen filling due to a reduction in the abomasal emptying rate caused by pectin viscosity [30]. Furthermore, feeding CiP has been shown to reduce DMI [32,33]. It has been suggested that pectin may interfere with ruminal fermentation (i.e., decreasing ruminal proportions of propionic acid while increasing acetic and butyric acids), resulting in greater rumen fill, while the increased density of CiP after hydration in the rumen may also help explain the observed effects on DM intake [12,32].

### 4.2. Nutrient Digestibility

Published data on nutrient digestibility with the use of citrus byproduct as a replacement for corn meal in dairy cows are inconsistent. Dineen et al. [34] reported reduced DM and NDF digestibility with the inclusion of CiP in the ration. Wing et al. [35] also observed continuous decreases in DM, OM, and ADF digestibility in cows fed 60, 120, or 180 g/kg DM of citrus molasses distiller’s soluble compared to corn-based rations. In contrast, Santos et al. [32] observed an increase in nutrient digestibility in cows fed CiP. Allam et al. [36], who substituted yellow corn grain in the ration with orange juice byproduct (peel, pulp and seeds) at four inclusion levels (0, 65, 135 and 195 g/kg DM), found no effect on digestibility. Similarly, Karimi et al. [28] found no differences in nutrient digestibility between rations containing 86.5 or 173 g of CiP per kg of DM. Corea et al. [12] conducted a meta-analysis with 14 studies in dairy cows and detected high between-study variability (I2 = 83.0%) with no significant differences (*p* = 0.71) in digestibility when dehydrated CiP (115 ± 5.44 g/kg diet DM) was used to replace CM.

Nutrient digestibility was reduced by the use of CiP, with the greater differences in ATTD observed in ADF and NDF (3.1 and 2.7 g/100 g, respectively), while for the CP ration, it was only 0.5 g/100 g. Based on this observation, it is hypothesised that the reduction in ATTD may be attributed to the carbohydrate content and type of the rations. CiP had 170 g/kg more NDF and 175 g/kg more ADF than CM (Table 1), which, as previously mentioned, was reflected in the overall diet composition. It should be noted that NDF content of the tropical forages (Table 1) and the forage-to-concentrate ratio used in this study led to dietary NDF concentrations that exceeded NRC [17] recommendations for high-yielding dairy cows. In the CiP ration, 8.28 g/100 g of the NDF and 10.62 g/100 g of the ADF were contributed by CiP, whereas in the CM ration, 5.63 g/100 g of the NDF and 2.43 g/100 g of the ADF were contributed by CM. NDF digestibility is known to be a predictor of total forage digestibility [37]. Increasing NDF concentration decreases overall diet digestibility [29,38], while hemicellulose has higher digestibility than cellulose in both legumes and grasses [39].

The substitution of 120 g/kg DM of CM for CiP in diets is equivalent to approximately 90 g/kg less starch and 48 g/kg more pectin. This could contribute to a decrease in diet nutrient digestibility, likely for a reduction in non-fibre carbohydrates (NFC); additionally, starch has a slightly higher ruminal rate and extent of degradability than pectin [40].

Pectins are rapidly soluble in water; their branched structure is composed of galacturonic acid; their lignin content up to 130 g/kg [17]. These characteristics cause pectins to undergo rumen fermentation more rapidly than cellulose and hemicellulose, generating primarily acetate and butyrate, with lower methane production compared to other fibres or starch sources [41]. On the other hand, the inclusion of pectin has been shown to reduce the rate of starch fermentation both in vitro [42] and in vivo [30]. In line with these findings, Culbertson et al. [43] reported a 4.2 g/100 g and 4.3 g/100 g higher DM and OM digestibility when comparing a high-NDF, low-starch diet (202 g/kg starch) with a low-NDF, high-starch diet (252 g/kg starch) in mid-lactation dairy cows.

### 4.3. Milk Production and Milk Composition

In this study, a 1 kg decrease in MY was observed when replacing corn meal with CiP. This result could be explained by a lower nutrient intake, since reducing DMI decreases MY [44]. This, combined with the lower nutrient digestibility observed in cows fed CiP, leads to a decreased supply of energy and amino acids available for milk synthesis. Solomon et al. [45] used CiP as a corn grain substitute in TMR fed to high-producing dairy cows and found that the cows receiving the CiP diet had lower DMI but similar MY compared with cows fed a high-corn diet, while Broderick et al. [29] concluded that feeding the pectin-rich carbohydrate source CiP altered ruminal fermentation and reduced both DMI and MY in cows. The concentration of NDF in the ration is negatively associated with DMI and MY [46]. However, regardless of the dietary NDF concentration, increased in vitro degradation has been associated with improved intake and production in early-lactation cows [47,48]. Therefore, the lower NDF content and higher digestibility of nutrients—including NDF—observed in CM rations in our experiment help explain the higher MY obtained with this treatment.

The effects of feeding CiP on MY in dairy cows have been shown to depend on both dietary factors (e.g., CiP level inclusion) and animal factors. The inclusion of CiP in the rations, at the expense of corn grain, resulted in linear decreases in MY. Steyn et al. [49] observed that decreases in MY for a 333, 666, and 1000 g/kg replacement of corn grain with CiP compared with zero replacement were 2.13, 2.27, and 3.23 kg/d, respectively. This is consistent with Ebrahimi et al. [50], who also observed reductions in MY with higher CiP inclusions, and with Hartinger et al. [51], who used meta-analysis to study the effect of dietary inclusion of CiP on MY in dairy cows at zero (0 g/kg), low (0–100 g/kg), medium (100–200 g/kg), or high (>200 g/kg) inclusion levels. They found that high CiP levels reduced DMI and MY, although milk had a higher fat concentration.

Results of a meta-analysis by Corea-Guillen et al. [12] with 44 trials showed an average reduction (*p* < 0.05) of 0.71 kg milk/d with the inclusion of CiP in dairy cow rations. Using a meta-regression approach, they also detected that the magnitude of the effect size on MY was negatively related to the amount of CiP included. Furthermore, a subgroup analysis showed that cows with high MY levels (>30 kg/d) experienced greater reductions in MY compared with the medium (20–30 kg/d) and low (<20 kg/d) groups. According to these results, they recommended that the amount of CiP in rations for high-producing cows should be kept below 30 g/kg DM.

Replacing CM with CiP can modify the ruminal microbiota (RM) due to the inclusion of pectin, resulting in a lower amount of energy for milk synthesis [29]. The ruminal microbiota differs between animals fed pectin-rich rations and those fed starch-rich rations [52] or in vitro cultures with substrates rich in pectin or starch [53]. The main pectinolytic species utilise a metabolic pathway other than glycolysis, which increases the molar concentration of acetate and produces less energy [6,54]. In vivo studies have shown that the VFA molar ratio is altered, with propionic acid decreasing while acetic and butyric acids increase in CiP rations compared to CM rations [12].

The decrease in milk nutrient production (CP, lactose, fat, total solids) observed in this study can be mainly explained by the reduction in MY in the CiP cow group. Furthermore, CP, lactose, and milk solid concentrations were also lower in CiP cows. Milk protein was affected by inclusion of citrus pulp in the diet due to the lower energy utilisation, as can be inferred from the lower digestibility of the ration [55]. Cabrita et al. [56] reported a reduction in milk nutrient concentrations when CM was replaced by CiP; they also observed that plasma glucose and insulin concentrations were lower in cows fed low-starch rations. Blood glucose is essential for mammary lactose synthesis and, therefore, for milk synthesis [57]. When the dietary starch supply is low, the utilisation of some amino acids for gluconeogenesis increases [58], reducing their availability for milk synthesis. Hence, feeding CiP to early and mid-lactation cows could be inadequate for milk yield.

### 4.4. Nitrogen Balance and Microbial Protein Synthesis

Nitrogen intake was lower (by 12 g/d) in cows fed the CiP diet compared with cows on the CM ration (Table 5), which could be related to the lower N output in urine and milk in the CiP cows. However, in the results expressed as a proportion of N intake (g N/100 g N), more N was excreted in the faeces and less was directed towards milk and urine in CiP cows. This result may also be related to the lower ATTD of protein in the same group of cows. It has been reported that the abomasal infusion of pectin decreases NDF digestibility and increases faecal N excretion in dairy cows [30].

Regarding N use in milk, it was reported that diets based on Cip had lower efficiency compared to those based on corn silage, which is rich in starch and thus provides a key source of fermentable energy for the rumen microbial population [59]. MUN can be used as an indicator of rumen N capture [60], as high MUN levels in dairy cows often indicate that a significant portion of the dietary protein is not being efficiently converted into microbial protein by ruminal microbes. Zhao et al. [61] recently reported that the NFC/CP ratio is highly correlated with MUN values; in addition, there is a negative correlation between the ingested N/milk N ratio and MUN.

In the current study, rations had the same CP concentration; however, N intake as well as CP digestibility were higher in cows on the CM ration, which could partially explain the higher urinary N output in this group, while the higher N efficiency in milk and lower MUN value could be related to the higher energy availability from starch compared with pectin in the CiP ration. As already mentioned, starch provides a rapid source of energy in the rumen for microbial N uptake. This can be confirmed by our observation of higher microbial protein synthesis and microbial efficiency in CM cows. Hristov et al. [62] reported that a higher intake of fermentable carbohydrates (starch or sucrose) increases microbial uptake of ammoniacal nitrogen released in the rumen compared to NDF, therefore improving the efficiency of microbial N use also for milk production. All this evidence is consistent with the current experiment, where the carbohydrate source of energy caused changes not only in DMI and ATTD but also in MPS and, consequently, MY, with better performance observed in cows fed CM compared with CiP.

### 4.5. Economic Comparison

The cost of the CiP ration was USD 0.23/d lower than the CM ration. Furthermore, milk revenue was USD 0.77/d higher for the CM ration. Therefore, the income over feed cost (IOFC) was USD 0.54/d higher for CM cows. Citrus pulp is used as an energy source; its lower cost compared to grains such as corn allows for the formulation of more economical rations for goats [63], buffalo [64], and dairy cows [65]. The IOFC can be used to monitor profits by incorporating gross milk income and feed costs [66]. In a study of 95 dairy farms, Buza et al. [67] estimated daily IOFC and concluded that minimising feed cost per cow per day did not maximise IOFC; instead, intermediate levels of forage costs and higher total feed cost per cow per day resulted in higher milk production and, consequently, higher IOFC. This suggests that optimal ration formulation, rather than lowest-cost strategies, may be key to increasing milk production and IOFC. In the current experiment, our corn–CiP ration was slightly more economical than the CM ration (0.27 vs. 0.28 USD/kg DM), and despite the higher DMI in cows fed CM, they had greater MY and IOFC. This finding reflects the better ruminal nutritional value of CM compared with CiP in high-producing cows under tropical conditions.

## 5. Conclusions

Feeding CiP as a partial replacement for CM affected the nutrient intake, digestibility, microbial protein synthesis and nitrogen use efficiency of cows under the conditions of this study, leading to reduced milk nutrient concentrations and milk yield. However, CiP could be a practical and cost-effective alternative feed source for medium- or low-yielding cows or during the second part of lactation in tropical dairy systems where conventional ingredients such as maize are expensive or scarce. Furthermore, its use could reduce producers’ dependence on a commodity subject to significant price and demand fluctuations, thereby mitigating economic vulnerability. Further research is needed to determine the optimal inclusion level and assess its effects on rumen fermentation, metabolism and productive performance.

## Figures and Tables

**Table 1:** Chemical composition (g/kg) of forages and main energy sources used in rations for the trial.

Nutrient	Corn Silage	Vigna Hay	Napier Grass	Corn Meal	Citrus Pulp
Dry matter	265 ± 14	866 ± 12	173 ± 16	889 ± 4.0	892 ± 5.8
Organic matter	911 ± 8.3	870 ± 9.6	860 ± 17	987 ± 1.8	917 ± 1.8
Crude protein	106 ± 6.5	135 ± 31	110 ± 25	82.4 ± 3.4	64.7 ± 3.4
Neutral detergent fibre	565 ± 20	534 ± 47	627 ± 38	106 ± 3.5	278 ± 3.9
Acid detergent fibre	315 ± 9.0	395 ± 42	370 ± 33	25.3 ± 3.8	201 ± 3.8

**Table 2:** Ingredients and composition of rations for dairy cows.

Ingredients, g/kg DM	CM	CiP	USD/kg as Feed
Corn meal	200	80	0.29
Citrus pulp	---	120	0.23
Soybean meal	139	146	0.39
Wheat bran	67	61	0.25
Cane molasses	56	56	0.14
Bypass fat	13.8	13.8	1.43
Sodium bicarbonate	7.1	7.1	0.46
Calcium carbonate	6.6	6.6	0.04
Mineral salt	5.3	5.3	2.31
Yeast *	2.2	2.2	1.60
Sodium chloride	2.0	2.0	0.10
Magnesium oxide	1.0	1.0	1.19
Corn silage	250	250	0.06
Vigna Hay	150	150	0.17
King grass	80	80	0.02
Swazi hay	20	20	0.02
Composition **			
Dry matter, g/kg	44.0	43.4	
Crude protein, g/kg DM	170	170	
ME, MJ/kg DM ***	10.1	9.9	
NDF, g/kg de DM	376	403	
ADF, g/kg DM	208	227	

* Diamond V™ (Cedar Rapids, IA, USA). ** According to laboratory analysis. *** According to balancing in the CPM Dairy V3 (CPM Dairy, version 3.0.10; Cornell University, Ithaca, NY, USA; University of Pennsylvania, Kennett Square, PA, USA; and William H. Miner Agricultural Research Institute, Chazy, NY, USA).

**Table 3:** Daily intake and apparent total tract digestibility (ATTD) of nutrients in dairy cows fed rations based on corn meal (CM) or citrus pulp (CiP) as main energy sources.

				*p* Value	
Item	CM	CiP	SEM	Tx	Carry Over
**Intake**					
Dry matter (DM, kg)	19.9	19.5	0.16	<0.01	0.18
Organic matter (OM, kg)	17.9	17.4	0.14	<0.01	0.19
Digestible OM (DOM, kg)	12.3	11.7	0.12	<0.01	0.09
Crude protein (CP, g)	3.43	3.35	0.30	<0.01	0.84
Neutral detergent fibre (NDF, kg)	7.39	7.63	0.65	<0.01	0.03
Acid detergent fibre (ADF, kg)	4.11	4.24	0.48	0.14	0.02
**ATTD (g/100 g)**					
DM	65.5	63.2	0.77	<0.01	0.03
OM	69.1	66.5	0.83	0.04	0.08
CP	70.4	69.9	0.76	0.02	0.03
NDF	50.4	47.7	1.18	<0.01	<0.01
ADF	49.9	46.8	1.39	<0.01	0.01

SEM, standard error of the mean.

**Table 4:** Milk composition and nutrient production in cows fed rations based on corn meal (CM) or citrus pulp (CiP) as main energy sources.

				*p* Value	
Item	CM	CiP	SEM	Tx	Carry Over
Milk yield (kg/d)	23.7	22.7	0.66	<0.01	0.18
Milk (kg)/Feed (kg DM)	1.20	1.16	0.04	0.09	0.10
**Milk composition**					
Milk fat (g/kg)	38.5	38.2	1.18	0.54	0.24
Milk protein (g/kg)	31.1	30.1	0.47	<0.01	0.03
Milk lactose (g/kg)	46.4	46.4	0.43	0.88	0.28
Milk total solids (g/kg)	124	123	1.60	0.01	0.22
MUN (mg/dL)	14.0	14.6	0.55	0.03	0.41
**Nutrient production**					
Milk fat (kg/d)	0.91	0.86	0.03	<0.01	0.85
Milk protein (kg/d)	0.73	0.67	0.02	<0.01	0.85
Milk lactose (kg/d)	1.09	1.05	0.03	0.02	0.11
Milk total solids (kg/d)	2.94	2.78	0.07	<0.01	0.36

SEM, standard error of the mean; MUN, milk urea nitrogen.

**Table 5:** Nitrogen (N) balance and microbial protein synthesis in dairy cows fed rations based on corn meal (CM) or citrus pulp (CiP) as main energy sources.

				*p* Value	
Item	CM	CiP	SEM	Tx	Carry Over
**N balance**					
N Intake (g/d)	550	538	9.81	<0.01	0.89
Faecal N (g/d)	178	184	4.67	0.17	0.41
Urinary N (g/d)	262	240	4.98	<0.01	0.42
Milk N (g/d)	118	109	3.25	<0.01	0.80
Faecal N (g/100 g N Intake)	32.5	34.4	0.87	<0.01	0.37
Urinary N (g/100 g N Intake)	47.8	44.8	1.10	0.03	0.73
Milk N (g/100 g N intake)	21.6	20.3	0.70	<0.01	0.82
**Microbial protein synthesis and efficiency**					
Purine derivatives excretion (PD, mmol/d)	419	362	18.6	0.01	0.34
Microbial protein synthesis (g/d)	1842	1561	91.2	0.01	0.34
Microbial N/N Intake (g/g)	0.53	0.46	0.02	0.04	0.26
Microbial N/DOMI * (g/kg)	23.8	21.3	1.12	0.11	0.84

SEM, standard error of the mean; * DOMI, digestible organic matter intake.

**Table 6:** Economic comparison of feeding cows with rations based on corn meal (CM) or citrus pulp (CiP) as main energy sources.

				*p* Value	
Variable	CM	CiP	SEM	Tx	Carry Over
Feeding cost (US$/d)	5.51	5.28	0.10	<0.01	0.26
Milk income (US$/d) *	17.07	16.30	0.48	<0.01	0.18
Income over feed cost (US$/d)	11.57	11.03	0.50	0.03	0.13
Feed cost/ milk income	0.50	0.56	0.04	0.29	0.33

SEM, standard error of the mean. * Estimated based on daily production and a price of $0.72 per litre of milk.

## Data Availability

The original contributions presented in this study are included in the article. Further inquiries can be directed to the corresponding author(s).
